# Development and efficacy evaluation of remodeled canine parvovirus-like particles displaying major antigenic epitopes of a giant panda derived canine distemper virus

**DOI:** 10.3389/fmicb.2023.1117135

**Published:** 2023-02-27

**Authors:** Shan Zhao, Xinfeng Han, Yifei Lang, Yue Xie, Zhijie Yang, Qin Zhao, Yiping Wen, Jing Xia, Rui Wu, Xiaobo Huang, Yong Huang, Sanjie Cao, Jingchao Lan, Li Luo, Qigui Yan

**Affiliations:** ^1^College of Veterinary Medicine, Sichuan Agricultural University, Chengdu, China; ^2^Key Laboratory of Animal Diseases and Human Health of Sichuan Province, Chengdu, China; ^3^Chengdu Research Base of Giant Panda Breeding, Chengdu, China

**Keywords:** giant panda, canine parvovirus, canine distemper virus, virus-like particles, immunogenicity

## Abstract

Canine parvovirus (CPV) and Canine distemper virus (CDV) can cause fatal diseases in giant panda (*Ailuropoda melanoleuca*). The main capsid protein of CPV VP2 can be self-assembled to form virus-like particles (VLPs) *in vitro*, which is of great significance for potential vaccine development. In the present study, we remodeled the VP2 protein of a giant panda-derived CPV, where the major CDV F and N epitopes were incorporated in the N-terminal and loop2 region in two combinations to form chimeric VLPs. The reactivity ability and morphology of the recombinant proteins were confirmed by Western blot, hemagglutination (HA) test and electron microscopy. Subsequently, the immunogenicity of the VLPs was examined *in vivo*. Antigen-specific antibodies and neutralizing activity were measured by ELISA, hemagglutination inhibition (HI) test and serum neutralization test (SNT), respectively. In addition, antigen specific T cell activation were determined in splenic lymphocytes. The results indicated that the VLPs displayed good reaction with CDV/CPV antibodies, and the heterologous epitopes do not hamper solubility or activity. The VLPs showed decent HA activity, and resembled round-shaped particles with a diameter of 22–26 nm, which is identical to natural virions. VLPs could induce high levels of specific antibodies to CPV and CDV, shown by the indication of neutralizing antibodies in both VP2N and VP2L VLPs group. In addition, splenic lymphocytes of mice immunized with VLPs could proliferate rapidly after stimulation by specific antigen. Taken together, the CPV VP2 VLPs or chimeric VLPs are highly immunogenic, and henceforth could function as CPV/CDV vaccine candidates for giant pandas.

## 1. Introduction

Canine parvovirus (CPV) and canine distemper virus (CDV) are common pathogens of domestic and wild carnivores and have a worldwide distribution. In recent years, they have shown a remarkable ability to cross species barriers, resulting in a broad and expanding host range due to antigenic drift and strain diversity (Miranda and Thompson, 2016), which indicated that they are not only severe pathogens endangering domestic dogs, also threats to the health of wild animal populations. As an endemic animal listed as “Vulnerable” in the International Union for the Conservation of Nature (IUCN) in 2016, the giant panda (*Ailuropoda melanoleuca*) is often implicated in the disease and mortality caused by CPV and CDV (Qin et al., 2010; Feng et al., 2016).

CPV is a small non-enveloped, singe-stranded DNA virus belonging to the genus Parvovirus and family Parvoviridae. To date, many evidences confirmed that CPV is transmitted to giant pandas which leads to severe infection, including diarrhea, vomiting and water-like feces and even death (Guo et al., 2013). VP2 is the most abundant protein found on the capsid and the N-terminal domain has been shown to be an excellent target for the development of a synthetic peptide vaccine (Langeveld et al., 1994; Chandran et al., 2009). VP2 consists of 584 amino acid residues, forming an 8-strand antiparallel β-fold and 4-loop structure (Rueda et al., 1999). These structures surround each other to form a triple fibrinoid, which contains the major epitopes of VP2 protein and the binding sites of the virus to the host receptor (Tsao et al., 1991).

Canine distemper is a highly contagious and fatal disease of a large number of species, including wild species, domestic dogs, and exotic animals held in zoos and parks worldwide, which is characterized by encephalitis with demyelination, diarrhea and respiratory disorders (Origgi et al., 2012). CDV is a member of genus Morbillivirus within the family Paramyxoviridae, containing a non-segmented single-stranded negative-sense RNA. The F glycoprotein is located on the envelop and more reliable than the other CDV proteins (Li et al., 2015), which mediates fusion between the virus and infected cells. As the most abundant and highly immunogenic viral protein in nature, the N protein get exposed to the host immune system for the significant production of antibodies (Yuan et al., 2015) and specific cell-mediated immunity (Beauverger et al., 1993). The neutralizing epitopes of CDV are primarily presented on F and N proteins, and these epitopes can provoke a robust immune response and neutralizing antibody (Ghosh et al., 2001; Bi et al., 2017).

The prevention and control of either canine parvo or canine distemper depends on vaccine. However, with the increasing epidemic trend of the diseases in giant panda population, there is no vaccine that could be applied in giant panda for less immune effect and potential hazards of the traditional canine-specialized vaccine. Thus, it is of great significance to develop a safe and efficient vaccine for giant panda. Virus-like particles (VLPs) retain the antigenic epitopes and structural morphology of natural virus particles, and effectively present glycoproteins and other antigenic components on the surface of virus particles (Clayton et al., 2002). After being recognized by B cells, VLPs can up-regulate B cell signals and major histocompatibility complex (MHC) class II molecules, which helps to induce high titer specific antibodies (Guo et al., 2021). As a granular antigen, VLPs could simulate the *in vivo* infection process of wild virus, which can effectively activate antigen presenting cells (APC) including monocytes/macrophages and dendritic cells (DCs), and activate T cells through the recognition of MHC class I molecules (Kushnir et al., 2012). In addition, VLPs is considered to have adjuvant effect (Fontana et al., 2020), which could be ingested by DCs without adjuvant through phagocytosis, penetration and Toll-like receptor-mediated activation, by which promote the maturation and migration of DCs and induce strong cellular immune response (Ding et al., 2010).

Based on VLPs, chimeric VLPs have been generated through genetic modifications or chemical couplings (Lei et al., 2020). Some of the protein subunits of VLPs allows the minor replacement or insertion of heterologous antigen without affecting the assembly of VLPs, potentially facilitating the display of immunodominant epitopes of heterologous antigens on the surface of VLPs (Wei et al., 2018; Pattinson et al., 2019). Thus, VLPs can be used as transfer vehicles carrying foreign proteins or antigen epitopes to produce chimeric VLPs for bivalent or multivalent vaccines (Hu et al., 2016; Watanabe et al., 2020). Due to the high immunogenicity and safety, VLPs have aroused a wide attraction for development of new subunit vaccines. Currently, a variety of commercial VLPs vaccines have been developed (Warfield and Aman, 2011; Kang et al., 2012; Kushnir et al., 2012). It has been reported that CPV VP2 can be self-assembled *in vitro* to form VLPs with similar morphology and structure as natural viruses (Xu et al., 2014). The N terminal and Loop2 region of CPV VP2 belong to non-essential region during assemble of VLPs. Neither addition of short amino acid residues nor minor displacements in these insertion sites hinders the formation of VLPs, indicating the accessibility of the chimeric VLPs of CPV VP2 (Rueda et al., 1999; Feng et al., 2011).

In this study, VLPs displaying major CDV antigenic epitopes were constructed by fusing the essential B-cell epitopes and T-cell epitopes of CDV in two combinations on CPV VP2 of giant panda origin. Moreover, the antigenicity and immunogenicity of the VLPs were evaluated by mouse immunization so as to provide a new method for the development of multivalent subunit vaccines against CPV and CDV infection that could be applied in giant pandas.

## 2. Materials and methods

### 2.1. Virus strains and cells

CPV strain JX624771 and CDV strain WL01 were isolated from giant panda in Sichuan Province of China and cultured in Vero cells in our laboratory. Madin–Derby canine kidney (MDCK) cell line was cultured at 37°C in 5% CO2 atmosphere in Dulbecco’s modified Eagle’s medium (DMEM; Gibco, United States). Vero cells expressing canine signaling lymphocyte activation molecules (SLAM) were cultured in DMEM (Gibco, United States) supplemented with 10% fetal calf serum (Gibco, United States) containing 0.1 mg/ml Zeocin (Invitrogen, United States).

### 2.2. Clone of wild-type VP2 gene of CPV originated from giant panda

DNA template of VP2 gene was extracted from MDCK cell pellets infected with CPV using MiniBEST Viral DNA/RNA Extraction Kit Ver.5.0 (Takara, Japan). A pair of primers with *Xho I* and *Hind III* was designed according to DNA sequences on Genbank (DQ354068.1) and used to amplify the whole sequence of VP2 gene (Table 1). PCR was performed using Taq Plus DNA Polymerase (Vazyme, Nanjing, China) or PCR Hero™ (Foregene, Chengdu, China) in a Bio-Rad cycler (United States). The PCR procedure was as follows: denaturation at 95°C for 30 s; denaturation at 95°C for 15 s, annealing at 65°C for 15 s; and polymerization at 72°C for 130 s with 30 cycles; extension at 72°C for 10 min. PCR products were detected in 1% agarose gel and visualized under UV light. The VP2 gene was amplified and cloned into pMD19-T plasmid vector (TaKaRa, Japan) to generate a recombinant plasmid of pMD19-T VP2.

**Table 1:** VP2 primer sequences and amplicon size of CPV.

Primer	Sequence (5′-3′)	Amplicon
VP2 F	CCGCTCGAGAGTGATGGAGCAGTTCAACCAGACG	1773 bp
VP2 R	CCCAAGCTTTTAATATAATTTTCTAGGTGCTAGTTGAG	

Underlined showed the restriction endonuclease sites of Xho I and Hind III.

### 2.3. Design and constructs of chimeric CPV VP2

The CDV epitopes identified in previous studies were applied for construction of CPV VP2 chimeras, including a B cell epitope and a CTL epitope of CDV N protein, a Th epitope, a T cell epitope and a B cell epitope of CDV F protein (Beauverger et al., 1993; Obeid et al., 1995; Ghosh et al., 2001; Yi et al., 2016; Table 2).

**Table 2:** CDV antigenic epitopes for chimera construction of CPV VP2 VLPs.

Region	Epitopes	Position (aa)	Characteristic
N protein	LNFGRSYFDPA	352–362	B
N protein	YPALGLHEF	283–291	CTL
F protein	TAAQITAGIALHQSNLN	241–257	Th
F protein	LSEVKGVIVHRLEAV	402–416	T
F protein	INQSPDKLLTF	518–528	B

**Figure 1:**
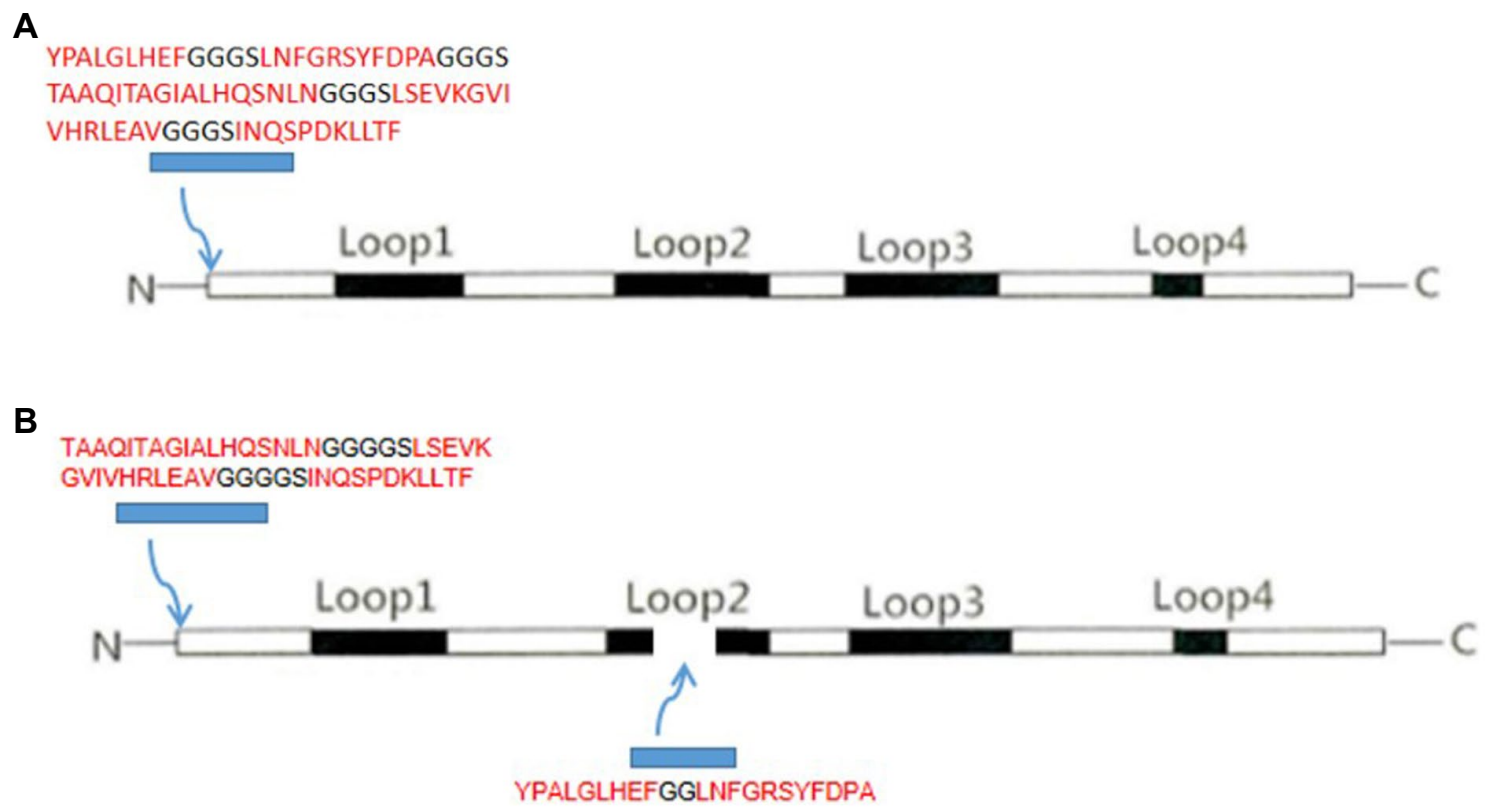
Schematic representation of **(A)** VP2N chimeric construct showing the insertion of CDV epitopes at the 5′ end of the CPV VP2 protein and **(B)** VP2L construct with CDV insertion epitopes both at the 5′ end and in the middle of CPV VP2 protein.

The chimeric protein included two combinations of VP2N and VP2L. VP2N was constructed with a N-terminus containing additional 78 amino acids ligated into the VP2 backbone. In brief, the epitope of CTL-B of CDV-N protein and the TH-T-B epitope of CDV-F protein were inserted into N terminal region of CPV-VP2 (Figure 1; Casal et al., 1995). As for VP2L, 53 additional amino acids including TH-T-B epitopes of CDV F protein were ligated into N terminus of VP2, and 22 amino acids containing CTL-B epitope of CDV N protein were inserted into the site utilizing the deletion of 218–233 AA at the Loop2 region of CPV VP2 (Figure 1; Rueda et al., 1999). In order to keep each epitope relatively independent, the epitopes were separated from one another by the linker with amino acid sequences GGGGS or GG.

The DNA sequences of each CDV epitope were optimized for expression in *Escherichia coli* with *in silico* analysis^1^, and were chemically synthesized and cloned into pMD19-T vector. Subsequently, the epitopes DNA sequences were conjugated into CPV VP2 gene using Splicing by Overlapping Extension (SOE) PCR. At last, VP2N/VP2L genes were cloned into pMD19-T vector with *Xho I* and *Hind III* site, respectively.

The plasmids of pMD19T-VP2/VP2N/VP2L were transformed into *E. coli* DH5α competent cells, and the *E. coli* containing plasmid of pMD19T-VP2/VP2N/VP2L were cultured in 5 ml LB broth containing 100 μg/ml of ampicillin at 37°C overnight. The recombinant plasmids were extracted with E.Z.N.A Plasmid Mini Kit (Omega, United States) and confirmed by *Xho I* and *Hind III* digestion. The inserts in the wild type and chimeric constructs were confirmed by Sanger DNA sequencing.

### 2.4. Expression and purification of CPV VP2/VP2N/VP2L in *Escherichia coli*

The full-length VP2/VP2N/VP2L genes were subcloned in frame into expression vector of pCold-TF (TaKaRa, Japan) at *Xho I* and *Hind III* sites, and the recombinant expression vectors pCold-TF-VP2/VP2N/VP2L were transformed into *E. coli* BL21 (DE3), respectively. After induction with 0.2 mmol/l IPTG, the cultures were collected and crushed by ultrasonic to confirm the presence and the distribution of the target recombinant protein by SDS-PAGE. Subsequently, the recombinant VP2/VP2N/VP2L protein was purified with Ni-NTA affinity chromatography (Qiagen, Germany). Since the purified recombinant protein had a large soluble Tag TF-Tag (46 kDa), 1a0 U Thrombin protease (Solarbio, China) was added to each 1 mg of purified recombinant protein for digestion at 4°C overnight. Lastly, the label and target proteins were separated by affinity chromatography.

### 2.5. Confirmation of the recombinant proteins by SDS-PAGE and Western blot

The purified recombinant proteins were transferred onto nitrocellulose filter membrane (NC membrane) after SDS-PAGE. Then NC membrane was blocked by 1% BSA for 2 h. After wash with Western washing buffer (TBS-T) for 3 times, the membranes were incubated overnight at 4°C with 1:5000 diluted mouse anti-CPV or anti-CDV monoclonal antibody (Jilin Wuxing Animal Health Care Co., Ltd., China). The membranes were washed by TBS-T again for 5 times and incubated with Horseradish Peroxidase (HRP)-conjugated goat anti-mouse secondary antibody (Sangon Biotech Co. Ltd., Shanghai) for 2 h. After another wash for 5 times with 5 min each time, electrochemiluminescence chemiluminescence (ECL) coloring solution (Sangon Biotech Co. Ltd., China) was added for color development under dark condition. The results were photographed with gel imaging system (Gel Doc™ XR^+^, Bio-Rad).

### 2.6. Self-assembly and characterization of CPV VP2 VLPs

After purification with Ni-NTA affinity chromatography, the recombinant protein was concentrated by ultrafiltration to remove the imidazole. The concentration of the recombinant protein was determined by the BCA Protein Assay Kit (Boster Biological Technology, United States). Subsequently, purified recombinant proteins of VP2/VP2L/VP2N were placed in a 14 kD dialysis bag with sufficient amount of dialysis buffer (50 mM NaH_2_PO_4_, 250 mM NaCl, 0.5%Triton X-100, 2 mM Dithiothreitol, pH8.0). The whole buffer system was placed on a magnetic agitator for slight stirring, and dialysis was conducted at 4°C for 16 h, during which the liquid was changed for 3 times. At last, 20 μl of the assembled sample was dropped on the copper net and placed for 2 min. The phosphotungstic acid solution was added for negative staining for 2–3 min, and the morphology of VLPs were observed by transmission electron microscope (TEM). Moreover, the hemagglutination test (HA) was used to detect the hemagglutination activity of the recombinant protein. The highest dilution concentration that could agglutinate 50% of porcine acetaldehyded erythrocytes (Guangzhou Hongquan Bio-tel Co. Ltd., China) was taken as the reading end point.

### 2.7. Immunization of mice with CPV VP2 VLPs

Fifty female BALB/c mice aged 6 weeks were randomly divided into 5 groups (n = 10). Group A, group B and group C were subcutaneously immunized with 50 μg of VP2, VP2L and VP2N recombinant protein, respectively. Group D was immunized with 100 μl CPV/CDV bivalent live-attenuated vaccine (Nobivac® Puppy DP, Intervet) as a positive control, and Group E injected with 100 μl PBS as negative control. Each group was boosted with the same dosage at 14-and 28-days post immunization (dpi). All animal experiments were conducted in compliance with protocols approved by Sichuan Provincial Laboratory Animal Management Committee (Permit Number: XYXK (Sichuan) 2019–187). The protocols for this experiment were performed according to the guidelines of the Ethics and Animal Welfare Committee (EAWC) of Sichuan Agricultural University. The mice were euthanized *via* exposure to carbon dioxide until complete cessation of breathing is observed for a minimum of 2 min by a trained technician during the experimental period as approved by EAWC.

### 2.8. Indirect ELISA for detection of specific antibodies in mice serum

At 0, 14, 28, 42, 56 days after first immunization, peripheral blood was obtained and serum was used to determine the antibody levels against CPV or CDV using an indirect ELISA method. Briefly, 96-well microtiter plates were coated with CPV JX624771 or CDV WL01 in 0.1 mol/l carbonate/bicarbonate buffer (pH 9.6) and incubated at 4°C overnight. After three washes in PBS-T, the plates were blocked with 250 μl PBS-T containing 1% BSA at 37°C for 2 h. After three washes in PBS-T, 100 μl of diluted mice serum (1:100) in PBS-T was added and incubated at 37°C for 1 h. Then mouse serum (1:100) with PBS-T containing 1% BSA was added, and plates were again incubated for at 37°C 1 h. After three washes in PBS-T, 100 μl diluted goat anti-mouse IgG peroxidase conjugate (Sangon Biotech Co. Ltd., China) in PBS-T containing 1% BSA at a 1:5, 000 dilution was then added for at 37°C for 1 h. The plates were washed three times, and the colorimetric reaction was developed using 100 μl tetramethylbenzidine substrate solution (Sangon Biotech Co. Ltd., China) at 37°C for 15 min. Color development was stopped with 50 μl of 2 N H_2_SO_4_, and OD was read at 450 nm.

### 2.9. Hemagglutination inhibition (HI) test

The CPV JX624771 strain was grown in MDCK cells and cell-free virus fluids were harvested. After inactivation by 0.025% β-propiolactone at 4°C for 24 h, CPV JX624771 was incubated at 37°C for 2 h and used as hemagglutination inhibition (HI) antigen. The HI test was performed at 14, 28, 42, 56 dpi as described previously (Senda et al., 1986). Briefly, the hemagglutination (HA) test was conducted in 96-well V-shaped microplates to determine the HA units of CPV JX624771. Twenty-five μL of serial 2-fold dilutions of inactivated CPV JX624771 with PBS was mixed with 25 μl of porcine erythrocyte suspension (Guangzhou Hongquan Bio-tel Co. Ltd., China) in each well and incubated at 4°C for 2 h. HA titers were determined and 4 HA units of inactivated CPV JX624771 were prepared. Serial 2-fold dilutions of heat inactivated serum were made in 25 μl of PBS in 96-well microplates and mixed with 25 μl CPV JX624771 containing 4 HA units. The mixture was incubated at 37°C for 30 min and 50 μl of porcine erythrocyte suspension was added for another incubation at 4°C for 1 h. The HI titer of serum was determined according to the reciprocal of the highest serum dilution which was a complete absence of HA.

### 2.10. Detection of neutralization antibody

The sera neutralizing antibody titers obtained from mice were determined by serum neutralization test (SNT) with a monolayer of Vero-SLAM cells at 56 dpi. Briefly, 50 μl of heat inactivated (56°C for 30 min) test sera were diluted serially 2-fold and mixed with equal volume of CDV WL01 strain (100 TCID_50_) in 96-well tissue culture plates. The plates were incubated at 37°C in 5% CO_2_ for 1 h. 100 μl of Vero-SLAM cells suspension (1 × 10^6^ cells/mL) was added and incubated at 37°C for 7 days for assessment of cytopathic effect (CPE). In addition, 100 TCID_50_ CDV virus diluent were set as positive control, and negative serums were added as negative controls. The highest dilution of serum showing complete inhibition of CPE was taken as the endpoint. The serum neutralization titer was expressed as the reciprocal of the final dilution of serum in the serum/virus mixture, which neutralized an estimated 100 TCID_50_ of the virus at 50% endpoint estimated according to the method of Kärber.

### 2.11. Spleen lymphocyte proliferation assay

Mouse spleens were removed under sterile conditions and ground through a sterile cuprous mesh (200 meshes) at 56 dpi. The spleen cells were immersed in RPMI 1640 medium (Gibco, United States) with 10% fetal calf serum, added to the lymphocyte separation medium (Sangon Biotech Co. Ltd., China), homogenized, and centrifuged at 1, 000 r/min for 10 min. Pellets were discarded; buoyant cells were washed thrice in RPMI 1640 medium with 10% fetal calf serum. The T lymphocytes in 96-well plates (1 × 10^5^ cells/well) were cocultured with 100 TCID_50_ CPV JX624771 or CDV WL01 in RPMI 1640 supplemented with 10% fetal calf serum, and maintained at 37°C in a humidified 5% CO_2_ atmosphere for 68 h. Meanwhile, negative control group was added with PBS. Each sample had 3 replicates. 3-(4,5-dimethylthiazol-2-yl)-2,5-diphenyl-2H-tetrazolium bromide (MTT) (5 mg/ml) was added to each well and incubated for 4 h at 37°C in 5% CO_2_. After culture for 4 h, the medium was gently removed, and 150 μl DMSO was added to each well. The plates were gently shaken to dissolve the crystals. The absorbance of each well was determined at 490 nm using a microplate reader, and the stimulatory index (SI) was calculated (SI = OD_490_ value of the stimulus group/OD_490_ value of the negative control group).

### 2.12. Statistical analyzes

Analysis of variance (ANOVA) and other statistical analysis were performed using GraphPad Prism version 5.0 (GraphPad Software, San Diego, CA, United States). Data was shown as the mean ± SEM, with the significant difference set at 0.05 (*p* < 0.05).

## 3. Results

### 3.1. Expression and purification of the recombinant proteins of CPV VP2/VP2N/VP2L

**Figure 2:**
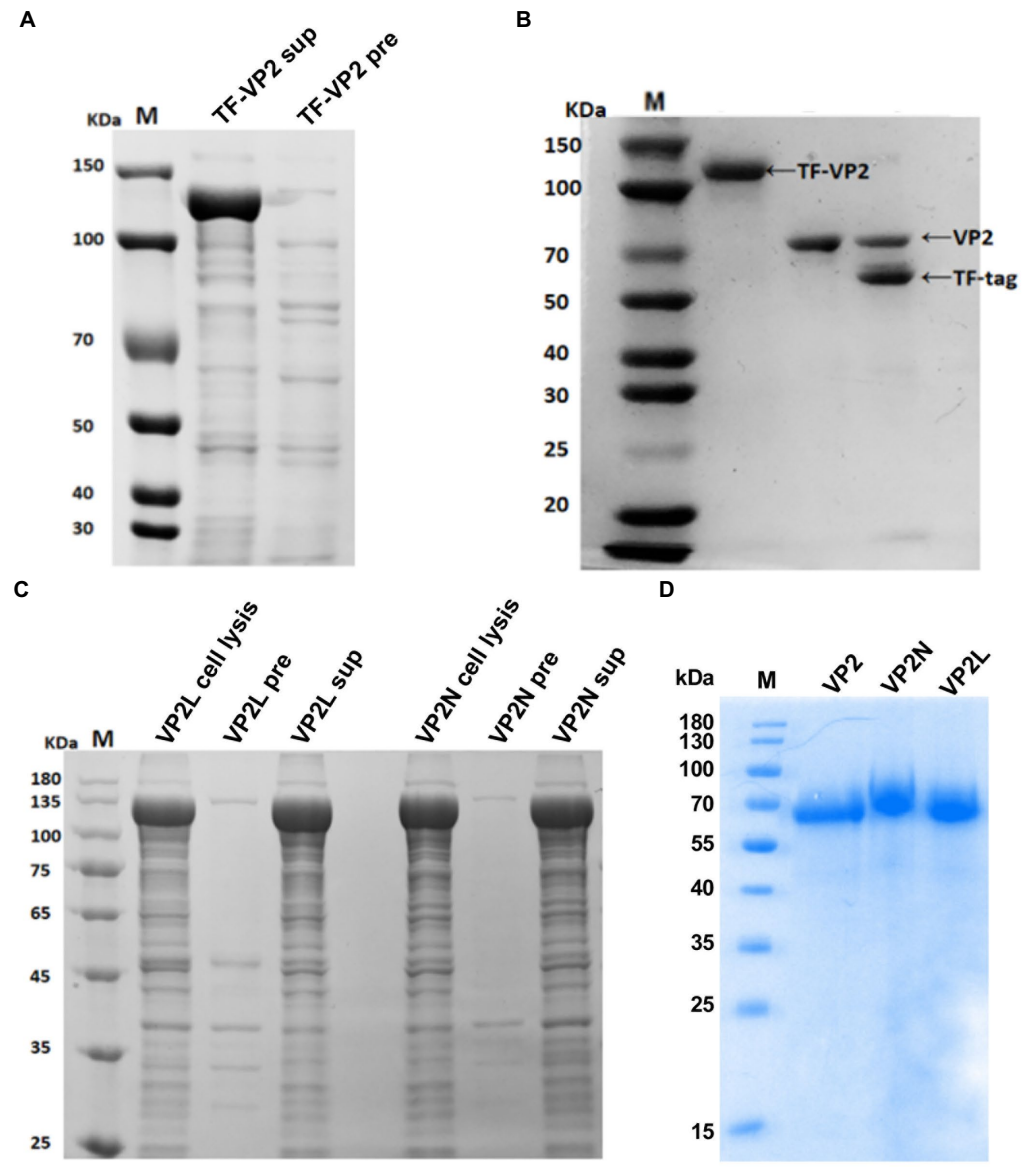
Expression and purification analysis of the recombinant protein of CPV VP2/VP2N/VP2L from *E. coli* by SDS-PAGE. **(A)** Expression of TF-VP2. Lane 1, TF-VP2 from supernatant (TF-VP2 sup); Lane 2, result from precipitate (TF-VP2 pre). **(B)** Enzyme digestion of TF-VP2 with Thrombin. Lane 1, TF-VP2 before digestion; Lane 2, VP2 protein after digestion and purification; Lane 3, TF-VP2 digested by Thrombin enzyme. **(C)** The soluble analysis of TF-VP2N/VP2L. Lane 1, pColdTF-VP2L whole cell (VP2L cell lysis); Lane 2, pColdTF-VP2L precipitate (VP2L pre); Lane 3, pColdTF-VP2L supernatant (VP2L sup); Lane 4, pColdTF-VP2N whole cell (VP2N cell lysis); Lane 5, pColdTF-VP2N precipitate (VP2N pre); Lane 6, pColdTF-VP2N supernatant (VP2N sup). **(D)** Recombinant protein after digestion and purification. Lane 1, VP2; Lane 2, VP2N; Lane 3, VP2L. Lane M represents the standard molecular weight markers.

To obtain the soluble protein expressed in *E. coli*, pCold TF DNA was used as expression plasmid in this study. Trigger factor (TF) is a prokaryotic ribosome-associated chaperone protein of 48 kDa which facilitates co-translational folding of nascent polypeptides, and tag removal from the expressed fusion protein. As a result, the recombinant protein TF-VP2 was successfully expressed in the supernatant (Figure 2A).

The supernatant after ultrasonic crushing was collected, and the recombinant protein in the supernatant was further purified by Ni-NTA affinity chromatography. The purified TF-VP2 was ultrafiltered to remove imidazole, and the appropriate amount of Thrombin was added to remove TF label overnight at 4°C by enzyme digestion. After overnight digestion, the solubilizing label of the recombinant protein TF-VP2 was removed by protease, and there was a distinct TF label at 56 kDa and a VP2 protein at 68 kDa (Lane 3; Figure 2B). Due to 6 × His tag on the removed TF tag, the protein solution after enzyme digestion was passed through Ni column again by Ni-NTA affinity chromatography to collect the flow fluid and soluble VP2 protein was obtained with high purity (Lane 2; Figure 2B). Furthermore, the recombinant proteins of both TF-VP2L and TF-VP2N were soluble, indicating that the soluble expression of the recombinant protein was not affected by CDV antigenic epitopes in the appropriate region of VP2 (Figure 2C). After purification by Ni-NTA affinity chromatography and Thrombin digestion, the pure recombinant protein without promoter label was obtained (Figure 2D).

### 3.2. Characterization of the recombinant protein with Western blot

**Figure 3:**
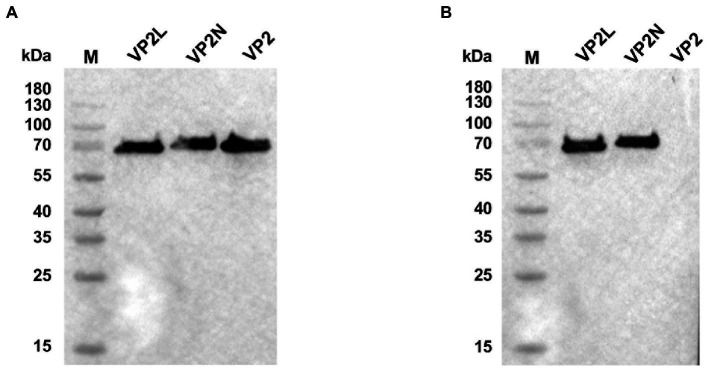
Western blot analysis of recombinant proteins of CPV VP2/VP2N/VP2L. **(A)** Using an anti-CPV monoclonal antibody as the primary antibody; **(B)** using an anti-CDV monoclonal antibody as the primary antibody.

After blotting, CPV or CDV monoclonal antibody was used as primary antibody for incubation and binding. Goat anti-mouse antibody labeled with HRP was used as secondary antibody. After ECL coloring, the results showed that specific bands at expected size of about 70 kDa were found on the NC membrane indicating that the purified recombinant proteins could be recognized by specific CPV antibody (Figure 3A). In addition, after incubation and binding with CDV monoclonal antibody as primary antibody, specific bands were also found on the NC membrane for the recombinant chimeric proteins, which indicated that chimeric epitopes constructs were successful and could react with specific CDV antibody (Figure 3B). The results above suggested that recombinant proteins of VP2/VP2N/VP2L displayed a single band with a molecular weight of 68 kDa/76 kDa/71.5 kDa, indicating that these proteins retained their specific immunoreactivity.

### 3.3. Assembly and characterization of VLPs

**Figure 4:**
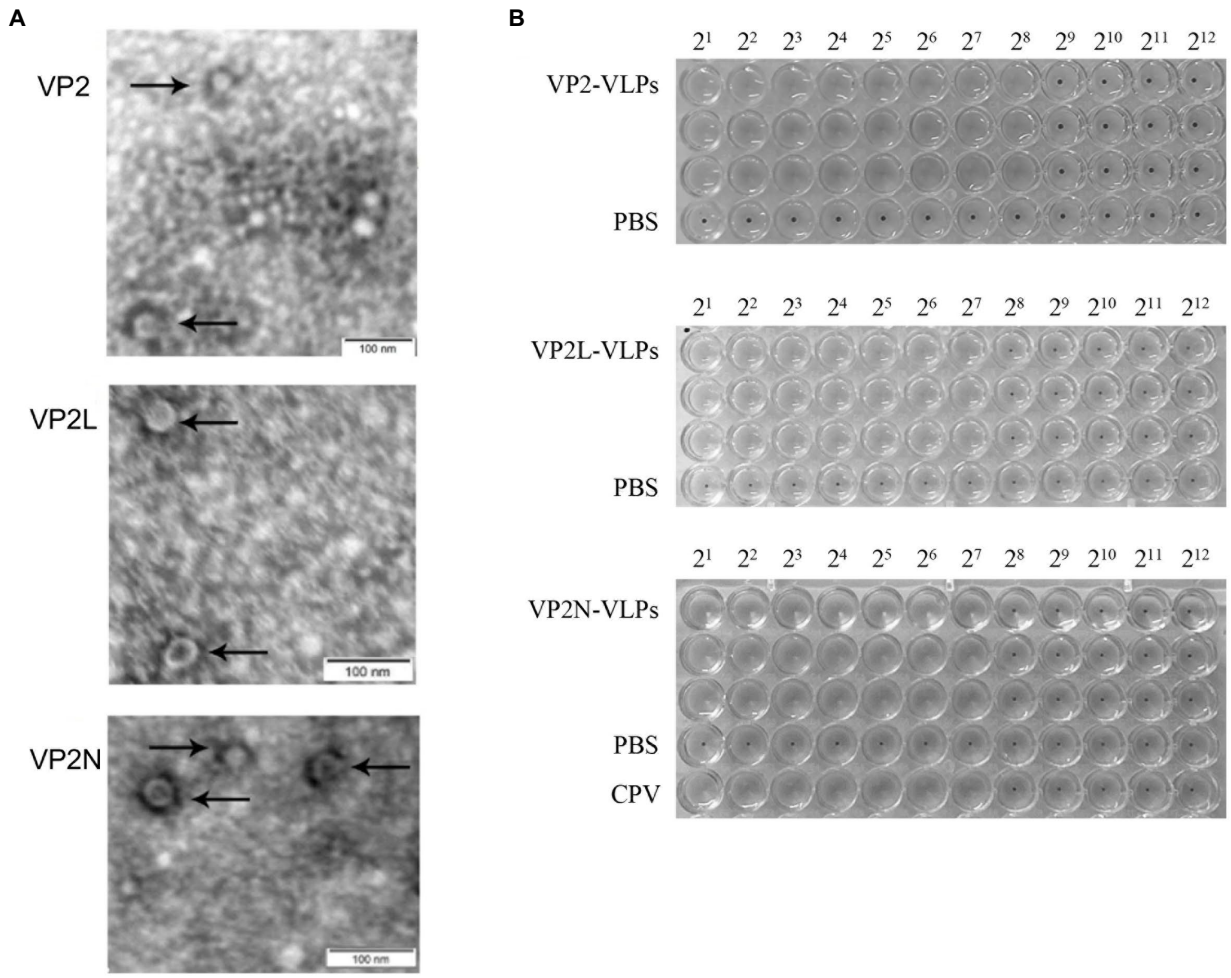
Characterization of wild-type and chimeric CPV VLPs by electron microscopy and HA test. **(A)** Electron micrographs of CPV VLPs, 80,000×; **(B)** Determination of HA titers of CPV VP2, VP2L and VP2N VLPs.

Electron microscopy revealed the presence of spherical particles of diameter in the range of 22 nm to 26 nm, which exhibited the same dimensions as natural CPV virions. The results indicated that the insertion of foreign epitopes into the appropriate position of VP2 protein does not affect the formation of VLPs, and the wild or chimeric VP2 proteins were capable of self-assembly into VLPs with diameters similar to CPV (Figure 4A).

HA test was conducted with 1% porcine erythrocyte suspension to detect the HA titer of the wild and chimeric VLPs in each group. Consequently, the HA titer of VP2 VLPs was 1:256, VP2L VLPs and VP2N VLPs were 1:128, indicating that the prepared VLPs had similar HA activity as wild CPV virions (Figure 4B).

### 3.4. Elisa detection of specific CPV/CDV antibodies in mice serum

**Figure 5:**
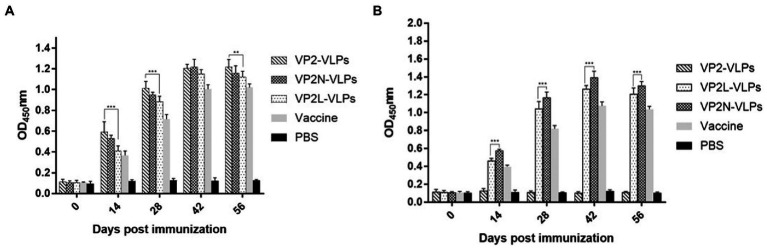
Detection of specific CPV/CDV antibodies in mice immunized with CPV VP2/VP2N/VP2L by indirect ELISA. Each group of 6-week-old Balb/c mice (*n* = 10) were immunized subcutaneously with 50 μg of VP2, VP2L, VP2N recombinant protein, 100 μl CPV/CDV live-attenuated vaccine and 100 μl PBS at 0, 14, 28 dpi. Blood was collected at 0, 14, 28, 42, 56 dpi and serum was obtained to determine the antibody titers of CPV/CDV. **(A)** Antibody titers of CPV in mice were determined by ELISA. **(B)** Antibody titers of CDV in mice serum were determined by ELISA. Data were analyzed using GraphPad 5.00. Asterisks indicate significant differences among the groups, as calculated by one-way ANOVA (**p < 0.01, ***p < 0.001).

Each mouse was bled to determine the titers of specific antibodies at 0, 14, 28, 42 and 56 dpi, respectively. The changes of CPV or CDV specific antibody levels in serum were detected by indirect ELISA, which showed that CPV or CDV specific antibodies were efficiently induced by immunizing with wild or chimeric VLPs. In contrast, no specific antibody was detected in negative control group injected with PBS (Figure 5). The results revealed that specific antibody levels in each immunization group increased continuously, reaching the peak at 42 dpi, and then maintained at a high level. At 42 dpi, there was no significant difference in antibody levels among VLPs immunization groups (*p* > 0.05), indicating that CPV specific antibodies could be produced in mice after VLPs immunization in each group, and the addition of CDV antigenic epitopes did not affect the production of specific antibodies to CPV (Figure 5A). Moreover, CDV specific antibodies in the two chimeric VLPs immunization groups also increased rapidly at 28 dpi, and the antibody level reached the peak at 42 dpi and slightly decreased at 56 dpi. In contrast, no CDV specific antibody was detected in PBS group and wild VP2 VLPs group. These results indicated that chimeric VLPs could effectively display the major epitopes of CDV, which could simultaneously induce CDV specific antibodies in mice (Figure 5B).

### 3.5. Specific HI antibody of CPV and neutralizing antibody against CDV in mice

**Figure 6:**
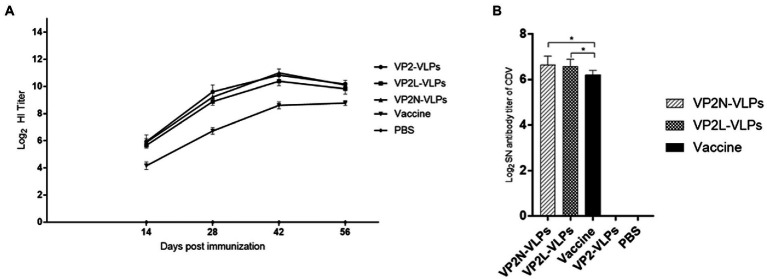
Determination of specific HI antibody titer against CPV at 14, 28, 42 and 56 dpi, and neutralizing antibody response to CDV *via* Vero-SLAM cells at 56 dpi after immunization with CPV VP2/VP2N/VP2L in mice. Each group of 6-week-old Balb/c mice (*n* = 10) were immunized subcutaneously with 50 μg of VP2, VP2L, VP2N recombinant protein, 100 μl CPV/CDV live-attenuated vaccine and 100 μl PBS at 0, 14, 28 dpi. **(A)** HI antibody titers of CPV in mice serum of each group at 14, 28, 42 and 56 dpi; **(B)** neutralizing antibody titers of CDV in mice of each group at 56 dpi. Data were analyzed using GraphPad 5.00. Asterisks indicate significant differences among the groups, as calculated by one-way ANOVA (*p < 0.05).

Specific HI antibody of CPV in mice serum was detected, and the change of HI antibody level was in harmony with that detected by indirect ELISA, which reached the peak at 42 dpi and tended to be stable after the third immunization (Figure 6A). Compared with the live attenuated vaccine group, there was significant difference (*p* < 0.05), and there was no significant difference among the VLPs immunization groups (*p* > 0.05).

SNT was applied to detect the titer of CDV neutralizing antibody in mice serum at 56 dpi in order to evaluate whether the chimeric proteins could effectively induce neutralizing antibody against CDV in mice. The results indicated that CDV specific neutralizing antibodies was produced in both the VLPs immunization group and the live attenuated vaccine group at 56 dpi (Figure 6A). CDV neutralizing antibody was not detected in VP2 VLPs group and PBS group. Compared with two chimeric VLPs, the average neutralizing antibody titer of VP2N VLPs group was 1:100.4, which was slightly higher than that of VP2L VLPS group (1:95.7), and there was no significant difference after analysis (*p* > 0.05). Moreover, the neutralizing antibody titer of live attenuated vaccine group was 1:74.0. There was significant difference between VP2N and VP2L groups at 56 dpi (*p* < 0.05; Figure 6B).

### 3.6. Detection of lymphocyte proliferation by MTT assay

**Figure 7:**
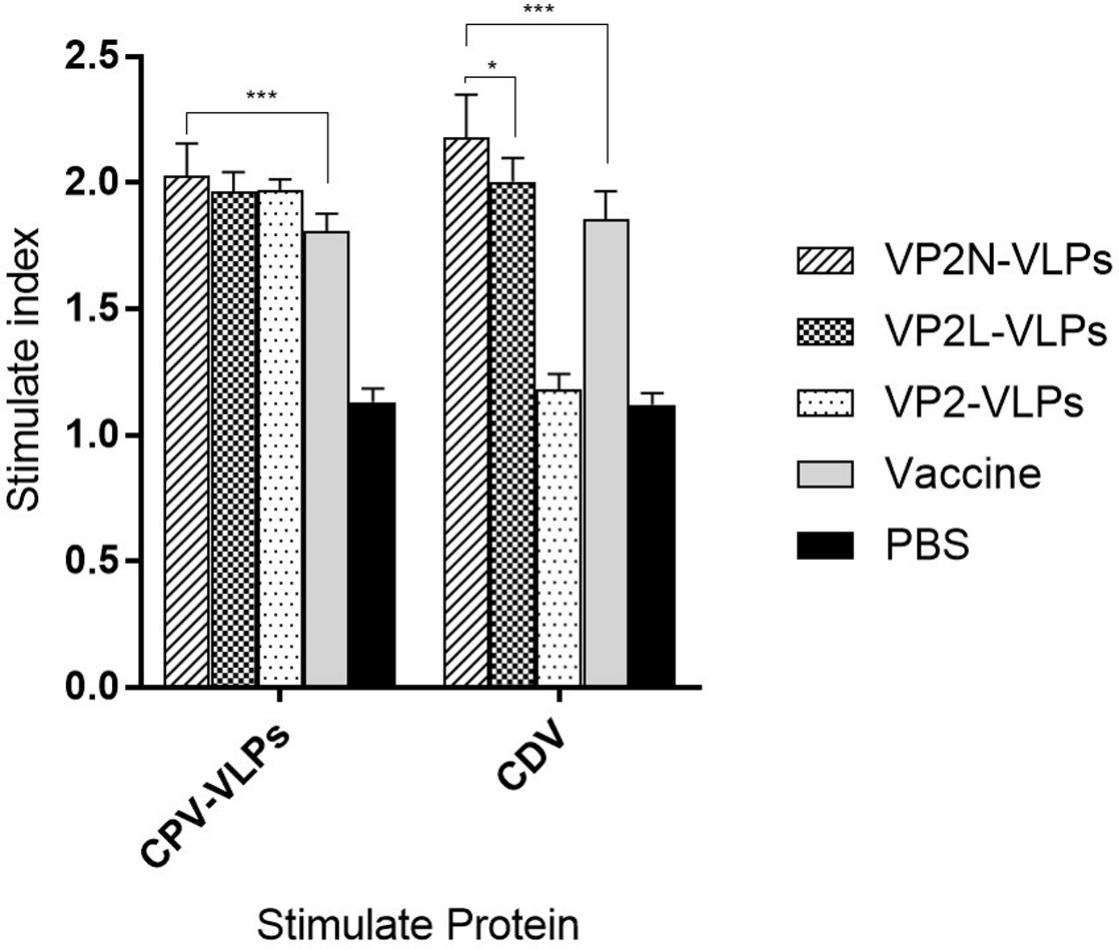
Lymphoid proliferation assay of mice with the stimulation of CPV or CDV at 56 dpi after immunization with CPV VP2/VP2N/VP2L in mice. Each group of 6-week-old Balb/c mice (*n* = 10) were immunized subcutaneously with 50 μg of VP2, VP2L, VP2N recombinant protein, 100 μl CPV/CDV live-attenuated vaccine and 100 μl PBS at 0, 14, 28 dpi. Data were analyzed using GraphPad 5.00. Asterisks indicate significant differences among the groups, as calculated by one-way ANOVA (*p < 0.05, ***p < 0.001).

CPV JX624771 or CDV WL01 virus were used to stimulate the splenic lymphocytes at 56 dpi, and PBS was used as the negative control. Splenic lymphocytes from mice in all immunization groups could proliferate under the stimulation of CPV, and the SI of VP2N VLPs group was significantly higher than live attenuated vaccine group (*p* < 0.01). However, there was no significant difference among three VLPs immunization groups (*p* > 0.05). With the stimulation of CDV, spleen lymphocytes in the chimeric VLPs group could respond to the stimulation and proliferate significantly. The SI of VP2N VLPs group was 2.18, which was significantly different from that of VP2L VLPs group (*p* < 0.05; Figure 7).

## 4. Discussion

The broad and expanding host range of CPV and CDV poses a great challenge for the control and eradication of these diseases. Giant pandas, especially captive ones, are susceptible to natural infection with CPV or CDV. At present, the prevention and control of the infections by these two viruses mainly rely on inactivated or live attenuated vaccines. However, there is no standard vaccine strategy for captive giant pandas in China. For example, most giant pandas at China Conservation and Research Center for the Giant Panda (Wolong Research Center) are not vaccinated, while at the Chengdu Research Base of Giant Panda Breeding are vaccinated with a canine live multivalent vaccine (including CDV, CPV, CAV-1, CCV, Canine parainfluenza virus (CPIV), and rabies virus). Although there is no evidence that live attenuated vaccines are directly related to the deaths of giant pandas currently, the possibility of subclinical disease may be significant. Neonatal infection from a vaccinated dam may result in Stunted Development Syndrome in giant panda (Janssen et al., 2006). Vaccination of cubs with modified live CDV vaccines may contribute to the gastrointestinal and respiratory illness (Qin et al., 2010). VLPs-based systems are reported to have better stability, fewer side effects, and, most importantly, they exhibit better uptake by macrophages, enterocytes, and DCs across the intestine and induce stronger immune response (Lin et al., 2014; Karandikar et al., 2017). In addition, the development of VLPs have led to more effective inactive vaccines that possess many advantages, including relatively accessible manufacturing technique, safety in production and use, and allowing display of heterologous epitopes (Chandran et al., 2009; Roose et al., 2013; Huber et al., 2017; Aston-Deaville et al., 2020).

It was obtainable to construct a chimera with an insertion of foreign epitopes between 218 and 233 amino acids in the middle of Loop 2 region of CPV VP2 without affecting the formation of VLPs *in vitro* (Hurtado et al., 1996; Yi et al., 2016). In this study, the structural plasticity of CPV VP2 protein was utilized to insert and fuse major B-cell epitopes, CTL epitope and T-cell epitopes of CDV at the N terminal and Loop2 regions to form chimeric CPV VPLs, so that CDV antigenic epitopes could be displayed on the surface of CPV VP2 VLPs. Consequently, both chimera constructs, including addition of 78 amino acids of the heterologous protein at the N-terminus and addition of 53 amino acids at the N-terminus together with insertion of 22 amino acids between the position of 218–233 amino acids residue of Loop 2 of CPV VP2, were successful without affecting the assembly process. In addition, the short linkers (GGGGS or GG) allowed the displaying of each individual epitopes on the surface of VLPs without interference on their functions. The fusion and display of epitopes on the CPV VLPs were confirmed by Western blot and HA assay, which indicated that the recombinant VLPs could react with specific CPV/CDV monoclonal antibodies and possess hemagglutination ability. Furthermore, electron microscopy indicated the formation of the wild or chimeric VLPs with particle diameters varying from 22 nm to 26 nm which was in accordance with previous researches that CPV VP2 expressed in *E. coli* could self-assemble into capsids *in vitro* (Rueda et al., 1999; Xu et al., 2014). These results demonstrated that the addition and insertion of chimeric peptides have no effect on the formation of CPV VP2 VLPs, which was consistent with previous results (Hurtado et al., 1996).

IgG plays a pivotal role in pathogen elimination at the systemic level. In this study, ELISA results showed that VLPs in each immunization group could induce a high level of CPV specific antibody, and the immune effect was similar to that of live attenuated vaccine. There was no significant difference in CPV antibody level between chimeric and wild VLPs, which indicated that heterologous epitopes had no influence on the production of CPV antibody. Moreover, the CDV specific antibodies produced by the VP2N group were slightly higher than those produced by VP2L group (*p* < 0.05), suggesting that the presenting effect of epitopes at the VP2-N terminal was better than that at the Loop2 region.

The HI titer of mice serum reflected the anti-CPV infection ability of animals after immunization. Mice serum immunized with wild and chimeric VLPs showed high HI titers against the filed isolates indicating a broad spectrum of protection against the isolates presently circulating in giant pandas. A strong positive linear correlation between HI activity and the amount of CPV-specific IgG antibodies was observed in mice immunized with wild or chimeric VLPs, suggesting that VLPs commonly induced CPV-specific HI antibodies. In addition, the effect of antibody level after immunization can be quickly calculated by measuring the HI titer. In this study, the HI titer in each immunization group showed the identical trend, and all reached the peak at 42 dpi (about 11 log2), which indicated that the VLPs could induce CPV specific neutralizing antibodies and had strong immunogenicity. Further, the induction of neutralizing antibody is essential for host defense against CDV (Ge et al., 2020; Bergmann et al., 2021). The fusion of CDV epitopes effectively induced CDV specific neutralizing antibodies. In contrast, VP2 VLPs induced only VP2-specific IgG. All the chimeras showed seroconversion following booster vaccination and SNT titer > 64 indicating the chimeric VLPs were responsible for the production of CDV neutralizing antibodies. The average neutralization antibody titer of VP2N group was 1:100.4, which was slightly higher than that of VP2L group (1:95.7) and live attenuated vaccine group (1:74.0), indicating that the chimeric CDV antigen epitopes were successfully constructed and displayed on the surface of CPV VP2 VLPS, which could stimulate a high level of CDV neutralizing antibody. However, immunogenicity in mice is not always predictive of immunogenicity in giant panda. Before moving into clinical trials for giant pandas, the results should be combined with data from higher order species to confirm vaccine responses.

It was previously reported that the lack of vaccine antigen-induced T cell activation could be overcome by genetic fusion of the antigen with T cell-stimulating peptides (Suzuki et al., 2018). Following this concept, we prepared a fusion protein of CTL, Th and T epitopes with CPV VP2. The results of spleen lymphocyte proliferation test showed that the lymphocyte proliferation in the VLPs groups were significantly higher than that in the negative control group after the stimulation of specific stimulants, further indicating that VLPs could induce both humoral immune response and cellular immune response. Compared with traditional subunit vaccine, CPV VLPs simulated the spatial structure of natural virus particles, which was more effective in antigen presentation and can induce stronger humoral and cellular immune responses. CPV VLPs allow the insertion of foreign epitopes without affecting its own structure and function. Through inserting heterologous epitopes into CPV VLPs, the immune effects can be expanded to achieve the characteristics of multivalent vaccines.

## 5. Conclusion

We prepared recombinant fusion proteins composed of CPV VP2 and essential antigenic epitopes of CDV to construct VLPs vaccine candidates against CPV and CDV of panda origin. VP2 VLPs induced both CPV-and CDV-specific immune responses in mice. Further, it resulted in the production of CPV-specific HI antibody against CPV and CDV-neutralizing IgG against CDV. These findings indicated that chimeric CPV VP2 VLPs are potential bivalent vaccine candidates against both CPV and CDV infection in giant pandas.

## Data availability statement

The original contributions presented in the study are included in the article/supplementary material, further inquiries can be directed to the corresponding author.

## Ethics statement

The animal study was reviewed and approved by the Ethics and Animal Welfare Committee (EAWC) of Sichuan Agricultural University.

## Author contributions

QY, XnH, and SZ: conceived and designed study, collected and complied, and analyzed data. ZY, YL, and QZ: statistical analyzes. SZ, XnH, YX, YW, JX, YH, RW, and XaH: drafted and edited manuscript. All authors contributed to the article and approved the submitted version.

## Funding

This study was funded by Chengdu Giant Panda Breeding Research Foundation (Grant No. CPF2017-25) and Natural Science Foundation of Sichuan Province (Grant No. 2022NSFSC1692 and 2022NSFSC1625).

## Conflict of interest

The authors declare that the research was conducted in the absence of any commercial or financial relationships that could be construed as a potential conflict of interest.

## Publisher’s note

All claims expressed in this article are solely those of the authors and do not necessarily represent those of their affiliated organizations, or those of the publisher, the editors and the reviewers. Any product that may be evaluated in this article, or claim that may be made by its manufacturer, is not guaranteed or endorsed by the publisher.

## Footnotes

^1^ http://www.bioinformatics.org/sms2/codon_usage.html

## References

[ref1] Aston-Deaville S. Carlsson E. Saleem M. Thistlethwaite A. Chan H. Maharjan S.. (2020). An assessment of the use of hepatitis B virus core protein virus-like particles to display heterologous antigens from *Neisseria meningitidis*. Vaccine 38, 3201–3209. doi: 10.1016/j.vaccine.2020.03.001, PMID: 32178907 PMC7113836

[ref2] Beauverger P. Buckland R. Wild T. F. (1993). Measles virus antigens induce both type-specific and canine distemper virus cross-reactive cytotoxic T lymphocytes in mice: localization of a common Ld-restricted nucleoprotein epitope. J. Gen. Virol. 74, 2357–2363. doi: 10.1099/0022-1317-74-11-2357, PMID: 7504072

[ref3] Bergmann M. Freisl M. Zablotski Y. Khan M. A. A. Speck S. Truyen U.. (2021). Prevalence of neutralizing antibodies to canine distemper virus and response to vaccination in client-owned adult healthy dogs. Viruses 13:945. doi: 10.3390/v13050945, PMID: 34065493 PMC8160937

[ref4] Bi Z. Wang Y. Pan Q. Xia X. Xu L. (2017). Development of CDV-specific monoclonal antibodies for differentiation of variable epitopes of nucleocapsid protein. Vet. Microbiol. 211, 84–91. doi: 10.1016/j.vetmic.2017.09.023, PMID: 29102126

[ref5] Casal J. I. Langeveld J. P. Cortés E. Schaaper W. W. van Dijk E. Vela C.. (1995). Peptide vaccine against canine parvovirus: identification of two neutralization subsites in the N terminus of VP2 and optimization of the amino acid sequence. J. Virol. 69, 7274–7277. doi: 10.1128/jvi.69.11.7274-7277.1995, PMID: 7474152 PMC189652

[ref6] Chandran D. Shahana P. V. Rani G. S. Sugumar P. Shankar C. R. Srinivasan V. A. (2009). Display of neutralizing epitopes of canine parvovirus and a T-cell epitope of the fusion protein of canine distemper virus on chimeric tymovirus-like particles and its use as a vaccine candidate both against canine parvo and canine distemper. Vaccine 28, 132–139. doi: 10.1016/j.vaccine.2009.09.093, PMID: 19818723

[ref7] Clayton R. F. Owsianka A. Aitken J. Graham S. Bhella D. Patel A. H. (2002). Analysis of antigenicity and topology of E2 glycoprotein present on recombinant hepatitis C virus-like particles. J. Virol. 76, 7672–7682. doi: 10.1128/JVI.76.15.7672-7682.2002, PMID: 12097581 PMC136371

[ref8] Ding F. X. Xian X. Guo Y. J. Liu Y. Wang Y. Yang F.. (2010). A preliminary study on the activation and antigen presentation of hepatitis B virus core protein virus-like particle-pulsed bone marrow-derived dendritic cells. Mol. BioSyst. 6, 2192–2199. doi: 10.1039/c005222a, PMID: 20820487

[ref9] Feng H. Liang M. Wang H. L. Zhang T. Zhao P. S. Shen X. J.. (2011). Recombinant canine parvovirus-like particles express foreign epitopes in silkworm pupae. Vet. Microbiol. 154, 49–57. doi: 10.1016/j.vetmic.2011.06.022, PMID: 21782359

[ref10] Feng N. Yu Y. Wang T. Wilker P. Wang J. Li Y.. (2016). Fatal canine distemper virus infection of giant pandas in China. Sci. Rep. 6:27518. doi: 10.1038/srep27518, PMID: 27310722 PMC4910525

[ref11] Fontana D. Marsili F. Etcheverrigaray M. Kratje R. Prieto C. (2020). Rabies VLPs adjuvanted with saponin-based liposomes induce enhanced immunogenicity mediated by neutralizing antibodies in cattle, dogs and cats. J. Virol. Methods 286:113966. doi: 10.1016/j.jviromet.2020.113966, PMID: 32905818

[ref12] Ge S. Xu L. Li B. Zhong F. Liu X. Zhang X. (2020). Canine parvovirus is diagnosed and neutralized by chicken IgY-sc Fv generated against the virus capsid protein. Vet. Res. 51:110. doi: 10.1186/s13567-020-00832-7, PMID: 32883344 PMC7468180

[ref13] Ghosh S. Walker J. Jackson D. C. (2001). Identification of canine helper T-cell epitopes from the fusion protein of canine distemper virus. Immunology 104, 58–66. doi: 10.1046/j.0019-2805.2001.01271.x, PMID: 11576221 PMC1783274

[ref14] Guo Y. Guo R. Ma Y. Chang W. Ming S. Yang G.. (2021). Chimeric virus-like particles of universal antigen epitopes of coronavirus and phage Qβ coat protein trigger the production of neutralizing antibodies. Curr. Top. Med. Chem. 21, 1235–1250. doi: 10.2174/1568026621666210618145411, PMID: 34145995

[ref15] Guo L. Yang S. L. Chen S. J. Zhang Z. Wang C. Hou R.. (2013). Identification of canine parvovirus with the Q370R point mutation in the VP2 gene from a giant panda (*Ailuropoda melanoleuca*). Virol. J. 10:163. doi: 10.1186/1743-422X-10-163, PMID: 23706032 PMC3680276

[ref16] Hu G. Wang N. Yu W. Wang Z. Zou Y. Zhang Y.. (2016). Generation and immunogenicity of porcine circovirus type 2 chimeric virus-like particles displaying porcine reproductive and respiratory syndrome virus GP5 epitope B. Vaccine 34, 1896–1903. doi: 10.1016/j.vaccine.2016.02.047, PMID: 26930366

[ref17] Huber B. Schellenbacher C. Shafti-Keramat S. Jindra C. Christensen N. Kirnbauer R. (2017). Chimeric L2-based virus-like particle (VLP) vaccines targeting cutaneous human papillomaviruses (HPV). PLoS One 12:e0169533. doi: 10.1371/journal.pone.0169533, PMID: 28056100 PMC5215943

[ref18] Hurtado A. Rueda P. Nowicky J. Sarraseca J. Casal J. I. (1996). Identification of domains in canine parvovirus VP2 essential for the assembly of virus-like particles. J. Virol. 70, 5422–5429. doi: 10.1128/jvi.70.8.5422-5429.1996, PMID: 8764053 PMC190499

[ref19] Janssen D. Morris P. Sutherland-Smith M. Greenberg M. Li D. Hu D.. (2006). Medical management of captive adult and geriatric giant pandas. Giant Pandas 353:376. doi: 10.1017/CBO9780511542244.016

[ref20] Kang S. M. Kim M. C. Compans R. W. (2012). Virus-like particles as universal influenza vaccines. Expert Rev. Vaccines 11, 995–1007. doi: 10.1586/erv.12.70, PMID: 23002980 PMC3513402

[ref21] Karandikar S. Mirani A. Waybhase V. Patravale V. B. Patankar S. (2017). “Chapter 10- Nanovaccines for oral delivery-formulation strategies and challenges” in Nanostructures for Oral Medicine. eds. Andronescu E. Grumezescu A. M. (Amsterdam, Netherlands: Elsevier), 263–293.

[ref22] Kushnir N. Streatfield S. J. Yusibov V. (2012). Virus-like particles as a highly efficient vaccine platform: diversity of targets and production systems and advances in clinical development. Vaccine 31, 58–83. doi: 10.1016/j.vaccine.2012.10.083, PMID: 23142589 PMC7115575

[ref23] Langeveld J. P. Casal J. I. Osterhaus A. D. Cortés E. de Swart R. Vela C.. (1994). First peptide vaccine providing protection against viral infection in the target animal: studies of canine parvovirus in dogs. J. Virol. 68, 4506–4513. doi: 10.1128/jvi.68.7.4506-4513.1994, PMID: 8207825 PMC236377

[ref24] Lei X. Cai X. Yang Y. (2020). Genetic engineering strategies for construction of multivalent chimeric VLPs vaccines. Expert Rev. Vaccines 19, 235–246. doi: 10.1080/14760584.2020.1738227, PMID: 32133886

[ref25] Li Z. Wang J. Yuan D. Wang S. Sun J. Yi B.. (2015). A recombinant canine distemper virus expressing a modified rabies virus glycoprotein induces immune responses in mice. Virus Genes 50, 434–441. doi: 10.1007/s11262-015-1169-x, PMID: 25764477

[ref26] Lin Y. Fengling L. Lianzhu W. Yuxiu Z. Yanhua J. (2014). Function of VP2 protein in the stability of the secondary structure of virus-like particles of genogroup II norovirus at different pH levels: function of VP2 protein in the stability of NoV VLPs. J. Microbiol. 52, 970–975. doi: 10.1007/s12275-014-4323-6, PMID: 25277406

[ref27] Miranda C. Thompson G. (2016). Canine parvovirus: the worldwide occurrence of antigenic variants. J. Gen. Virol. 97, 2043–2057. doi: 10.1099/jgv.0.000540, PMID: 27389721

[ref28] Obeid O. E. Partidos C. D. Howard C. R. Steward M. W. (1995). Protection against morbillivirus-induced encephalitis by immunization with a rationally designed synthetic peptide vaccine containing B-and T-cell epitopes from the fusion protein of measles virus. J. Virol. 69, 1420–1428. doi: 10.1128/jvi.69.3.1420-1428.1995, PMID: 7531779 PMC188728

[ref29] Origgi F. C. Plattet P. Sattler U. Robert N. Casaubon J. Mavrot F.. (2012). Emergence of canine distemper virus strains with modified molecular signature and enhanced neuronal tropism leading to high mortality in wild carnivores. Vet. Pathol. 49, 913–929. doi: 10.1177/0300985812436743, PMID: 22362965

[ref30] Pattinson D. J. Apte S. H. Wibowo N. Chuan Y. P. Rivera-Hernandez T. Groves P. L.. (2019). Chimeric murine Polyomavirus virus-like particles induce plasmodium antigen-specific CD8(+) T cell and antibody responses. Front. Cell. Infect. Microbiol. 9:215. doi: 10.3389/fcimb.2019.00215, PMID: 31275867 PMC6593135

[ref31] Qin Q. Li D. Zhang H. Hou R. Zhang Z. Zhang C.. (2010). Serosurvey of selected viruses in captive giant pandas (*Ailuropoda melanoleuca*) in China. Vet. Microbiol. 142, 199–204. doi: 10.1016/j.vetmic.2009.09.062, PMID: 19913371 PMC7117238

[ref32] Roose K. De Baets S. Schepens B. Saelens X. (2013). Hepatitis B core-based virus-like particles to present heterologous epitopes. Expert Rev. Vaccines 12, 183–198. doi: 10.1586/erv.12.150, PMID: 23414409

[ref33] Rueda P. Hurtado A. del Barrio M. Martínez-Torrecuadrada J. L. Kamstrup S. Leclerc C.. (1999). Minor displacements in the insertion site provoke major differences in the induction of antibody responses by chimeric parvovirus-like particles. Virology 263, 89–99. doi: 10.1006/viro.1999.9911, PMID: 10544085

[ref34] Senda M. Hirayama N. Yamamoto H. Kurata K. (1986). An improved hemagglutination test for study of canine parvovirus. Vet. Microbiol. 12, 1–6. doi: 10.1016/0378-1135(86)90035-0, PMID: 3727364

[ref35] Suzuki H. Hosomi K. Nasu A. Kondoh M. Kunisawa J. (2018). Development of adjuvant-free bivalent food poisoning vaccine by augmenting the antigenicity of *Clostridium perfringens* enterotoxin. Front. Immunol. 9:2320. doi: 10.3389/fimmu.2018.02320, PMID: 30356722 PMC6189403

[ref36] Tsao J. Chapman M. S. Agbandje M. Keller W. Smith K. Wu H.. (1991). The three-dimensional structure of canine parvovirus and its functional implications. Science 251, 1456–1464. doi: 10.1126/science.2006420, PMID: 2006420

[ref37] Warfield K. L. Aman M. J. (2011). Advances in virus-like particle vaccines for filoviruses. J. Infect. Dis. 204, S1053–S1059. doi: 10.1093/infdis/jir346 21987741 PMC3189993

[ref38] Watanabe S. Fujimoto Z. Mase M. (2020). Development of immunogenic chimeric virus-like particles based on bovine papillomavirus type 6. Vaccine 38, 7774–7779. doi: 10.1016/j.vaccine.2020.10.037, PMID: 33164801

[ref39] Wei S. Lei Y. Yang J. Wang X. Shu F. Wei X.. (2018). Neutralization effects of antibody elicited by chimeric HBV S antigen viral-like particles presenting HCV neutralization epitopes. Vaccine 36, 2273–2281. doi: 10.1016/j.vaccine.2018.03.036, PMID: 29576303

[ref40] Xu J. Guo H. C. Wei Y. Q. Dong H. Han S. C. Ao D.. (2014). Self-assembly of virus-like particles of canine parvovirus capsid protein expressed from *Escherichia coli* and application as virus-like particle vaccine. Appl. Microbiol. Biotechnol. 98, 3529–3538. doi: 10.1007/s00253-013-5485-6, PMID: 24413974

[ref41] Yi L. Cheng Y. Zhang M. Cao Z. Tong M. Wang J.. (2016). Identification of a novel canine distemper virus B-cell epitope using a monoclonal antibody against nucleocapsid protein. Virus Res. 213, 1–5. doi: 10.1016/j.virusres.2015.10.022, PMID: 26514066

[ref42] Yuan B. Li X. Y. Zhu T. Yuan L. Hu J. P. Chen J.. (2015). Antibody study in canine distemper virus nucleocapsid protein gene-immunized mice. Genet. Mol. Res. 14, 3098–3105. doi: 10.4238/2015.April.10.20, PMID: 25966074

